# A nonsense mutation in *C8orf37* linked with retinitis pigmentosa, early macular degeneration, cataract, and myopia in an arRP family from North India

**DOI:** 10.1186/s12886-023-02936-y

**Published:** 2023-05-11

**Authors:** Shiwali Goyal, Kabir Singh, Aashna Uppal, Vanita Vanita

**Affiliations:** 1 Department of Human Genetics, Guru Nanak Dev University, Amritsar, Punjab India; 2 Dr. Daljit Singh Eye Hospital, Amritsar, Punjab India

**Keywords:** Retinitis pigmentosa, Macular degeneration, C8orf37, Whole-exome sequencing, Nonsense mutation

## Abstract

**Objective:**

This study aimed at identifying the underlying genetic defect in a consanguineous autosomal recessive retinitis pigmentosa (arRP) (RP-1175) family having RP with early macular degeneration, cataract, and myopia.

**Methods:**

Whole-exome sequencing (WES) was performed on the DNA of the proband, and variants observed were validated in the rest of the affected and unaffected family members by Sanger sequencing. Different bioinformatics tools were applied to access the pathogenicity of the observed variant.

**Results:**

A nonsense mutation i.e., c.555G > A (p.Trp185Ter) in *C8orf37* in homozygous form, has been identified that segregated with the disease in the affected members. c.555G > A was absent in unaffected family members and in 107 ethnically matched controls, therefore ruling out its possibility of being a polymorphism.

**Conclusions:**

Present study identifies a nonsense mutation (c.555G > A) at codon 185 in *C8orf37* linked with arRP, early macular degeneration, posterior subcapsular cataract, and myopia. The identical mutation has previously been reported in a Pakistani family with isolated RP and in a Chinese family with RP and macular degeneration. This variable expressivity of the identified mutation c.555G > A in *C8orf37* in the analyzed Indian family may be attributed to the presence of the modifier alleles. Also, Trp185 might be a mutation hotspot in Asian arRP patients and in the future, p.Trp185Ter in *C8orf37* may be tested during initial screening in arRP cases especially belonging to a similar population.

**Supplementary Information:**

The online version contains supplementary material available at 10.1186/s12886-023-02936-y.

## Introduction

Retinitis pigmentosa (RP; OMIM: 26,8000) is an inherited retinal degenerative disease with a predictable prevalence of one in 1,798-5,260 individuals [1]. Approximately 1.5 million individuals are affected by RP worldwide [2]. RP patients initially suffer nyctalopia due to rod cell dysfunctioning followed by regular loss of peripheral and central vision due to dysfunctioning of cone cells [3]. RP exhibits all three Mendelian modes of inheritance i.e., autosomal dominant (adRP), autosomal recessive (arRP), and X-linked (xlRP) [2, 4]. xlRP is a relatively severest form of RP with early age-of-onset of disease and fast progression. Rare forms of inheritance such as mitochondrial, X-linked dominant, and digenic, although not frequent, however, are also reported in RP [4–6]. More than 130 genes have been linked with RP with ~ 90 genes being linked with isolated, non-syndromic RP and approximately 40 genes for syndromic RP (https://sph.uth.edu/retnet/sum-dis.htm*).*

Dysfunctioning of retinal pigment epithelium (RPE) leads to degeneration of rod and cone cells, followed by loss of vision and blinding disorders in humans such as age-related macular degeneration and retinitis pigmentosa [7]. Functionally, a large number of RP-linked genes encode proteins with diverse functions such as; phototransduction pathway (visual cascade and visual cycle) (*RHO*, *PDE6A*, *PDE6B*, *CNGA1*, *CNGB1*, *SAG*); vitamin A metabolism pathway (*ABCA4*, *RLBP1*, *RPE65*, *LRAT*, *RGR*); structural or cytoskeletal (*RDS*, *ROM1*, *FSCN2*, *TULP1*, *CRB1*, *ORP1*); signaling, cell-cell interaction, or synaptic interaction (*SEMA4A*, *CDH23*, *PCDH15*, *USH1C*, *USH2A*, *USH3A*, RP2); mRNA intron-splicing factors (*PRPF31*, *PRPF8*, *PRPF3*, RP9); intracellular proteins trafficking (*MYO7A*, *USH1G*); maintenance of cilia/ciliated cells (*BBS1*, *BBS2*, *ARL6*, *BBS4*, *BBS5*, *MKKS*, *BBS7*, *TTC8*, *RPGR*); pH regulator (*CA4*); phagocytosis (*MERTK*) and proteins with unknown function (*C8orf37*, *C2orf71*, *CERKL*, *IMPDH1*, *BBS10*) [2]. Stem cell and gene therapy approaches hold a lot of promise for the treatment of retinal dystrophies, yet no such effective treatments are in use till date. However, identifying genes and mutations is crucial for genetic diagnosis and prenatal diagnosis.

In the present study, an arRP family hailing from the Northern part of India having two members affected with RP in association with early onset macular degeneration, posterior subcapsular cataract, and myopia were analyzed. On the DNA sample of the proband whole-exome sequencing (WES) was performed followed by annotation of variants. WES data analysis revealed c.555G > A (p.Trp185Ter), a previously reported substitution in exon 6 of *C8orf37* (NM_177965.3) in homozygous form in the proband. On validation using Sanger sequencing, c.555G > A (p.Trp185Ter) substitution was co-segregating in homozygous form in both the affected members whereas four unaffected members of the family were heterozygous for this change. The observed nucleotide change was also not observed in 107 ethnically matched healthy controls, (free of any retinal disease) hence suggestive of excluding it as a polymorphism.

## Methods

The study was approved by the Institutional Ethics Committee (IEC) of the Guru Nanak Dev University (GNDU), Amritsar, India in accordance with the Declaration of Helsinki. From all the participants of the study written informed consents were obtained.

### Family description and clinical evaluation

**Fig. 1:**
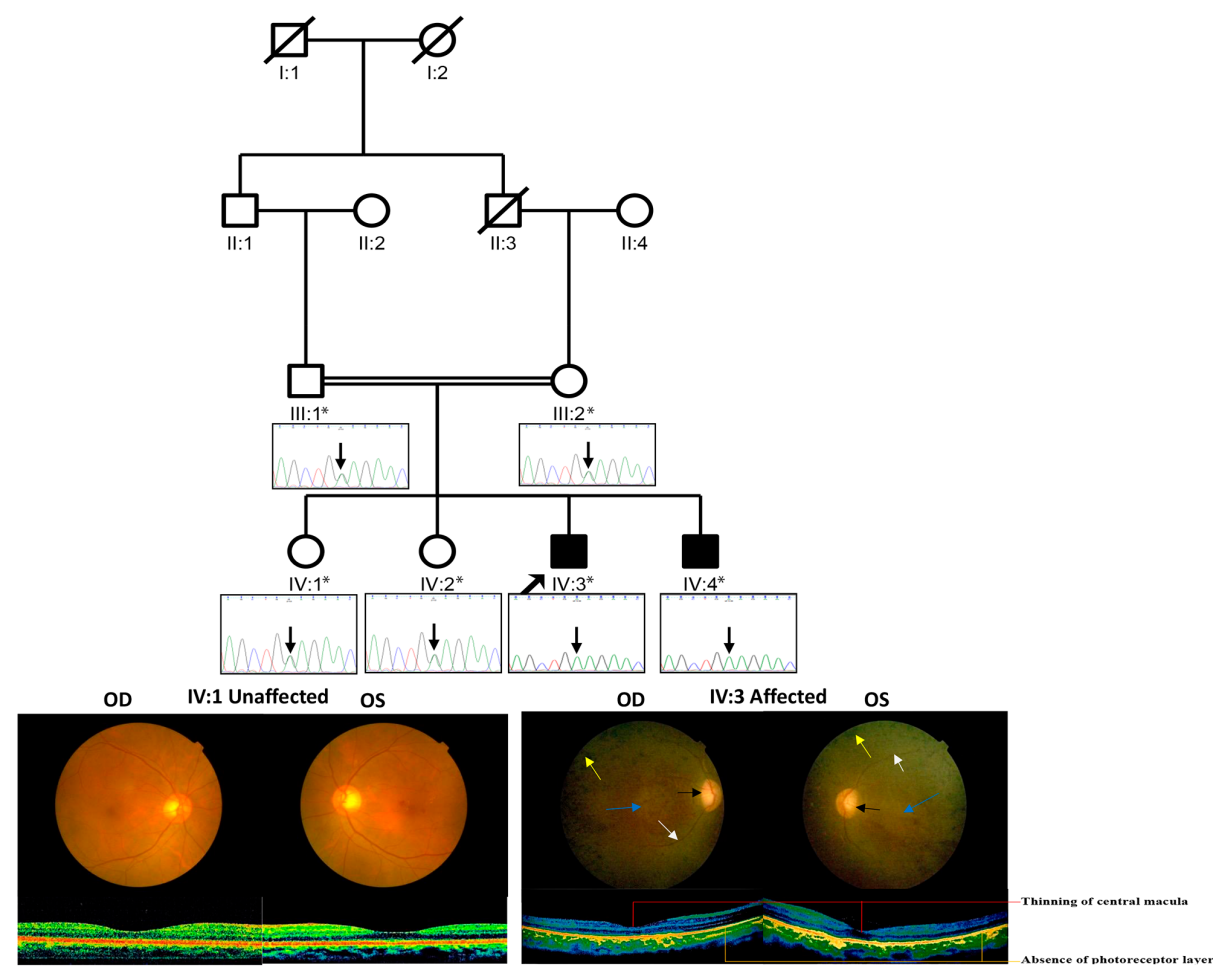
In the pedigree, filled and unfilled symbols represent affected and unaffected members, respectively. Squares indicate males, and circles as females. Arrow indicates the index case (proband). Diagonal lines through squares/circles represent deceased persons. Double horizontal line indicates consanguinity between individuals III: 1 and III: 2 (first cousins; parents of the proband). Symbols indicated with stars are family members who underwent ophthalmic examination and gave their blood samples for analysis. Fundus photographs of affected individual IV: 3 showed attenuated retinal blood vessels (marked by white arrows), waxy pallor of the optic disc (as indicated by black arrows), ‘bone spicules’ like pigmentation (marked by yellow arrows), and macular degeneration (as depicted by blue arrows). Fundus photographs of an unaffected individual (IV: 1) showed no sign of retinal degeneration. OCT photographs of the patient (IV: 3) indicated absence of photoreceptors layer (marked with yellow lines) and thinning of central macula (marked with red lines). OCT photographs of the unaffected sister (IV: 1) of the proband indicated normal retinal architecture. Electropherogram of a part of the reverse strand sequence of exon 6 of *C8orf37* in the affected and unaffected members of the family. Arrow indicated the base at which homozygous change c.555G > A occurred that resulted in p.Trp185Ter substitution in both the affected members (IV: 3 and IV: 4)

A 20-year-old male (IV: 3; proband) **(**Fig. 1**)** who visited Dr. Daljit Singh Eye Hospital (Dr. DSEH), Amritsar, Punjab, India, was diagnosed with nonsyndromic retinitis pigmentosa in association with early macular degeneration with myopia in both the eyes and posterior subcapsular cataract in the left eye. Detailed family history taken up to four generations using the criteria given by Bennett et al. [8], and careful pedigree investigation displayed this to be an autosomal recessive family with two members affected (IV:3 and IV:4) in a single sibship and their parents (III: 1 and III: 2) being first cousins. Ophthalmic examination including visual acuity testing (VA), intra-ocular pressure (IOP) measurement, and dilated fundus examination followed by fundus photography, carried out on six members (III: 1, III: 2, IV: 1, IV: 2, IV: 3, and IV: 4) of the family, confirmed two members (IV: 3 and IV: 4) to be affected by typical RP in association with maculopathy **(**Table 1**)**. Optical coherence tomography (OCT) testing could be performed on both the affected members (IV: 3 and IV: 4) and their unaffected sibling (IV: 1).

**Table 1:** Personal and ophthalmic details of clinically examined members of an arRP (RP-1175) family

Individual’s ID^a^	Sex	Age (yrs)	Aff/Unaff	Visual acuity	Age-of-onset of RP (yrs)	Refractive correction	Any associated anomaly	Fundus findings	OCT findings
III: 1	M	60	Unaff	6/18 (OD) 6/18 (OS)	-	-0.50D (OD) -0.75D (OS)	NA	No RP changes	-
III: 2	F	55	Unaff	6/12 (OD) 6/12 (OS)	-	-0.50D (OD) + 1.50D (OS)	NA	No RP changes	-
IV: 1	F	27	Unaff	6/6 (OD) 6/6 (OS)	-	-1.00D (OD) -1.25D (OS)	NA	No RP changes **(**Fig. 1**)**	OCT depicted normal retinal architecture **(**Fig. 1**)**
IV: 2	F	23	Unaff	6/6 (OD) 6/6 (OS)	-	-0.50D (OD) -0.50D (OS)	NA	No RP changes	-
IV: 3	M	20	Aff	< 6/60 (OD) 6/60 (OS)	10	-4.50D (OD) -5.50D (OS)	Posterior subcapsular cataract (L/E)	Bilateral arterial attenuation, optic disc pallor, bone spicules at the periphery, macular degeneration B/E **(**Fig. 1**)**	OCT revealed absence of photoreceptors layer **(**Fig. 1**)**
IV: 4	M	17	Aff	< 6/60 (OD) 6/60 (OS)	11	-10.00D (OD) -10.00D (OS)	Posterior subcapsular cataract (R/E)	Bilateral arterial attenuation, optic disc pallor, bone spicules at the periphery, macular degeneration B/E	OCT depicted absence of photoreceptors layer

M = male; F = female; Unaff = unaffected; Aff = affected; OD = oculus dexter; OS = oculus sinister; NA = no any; L/E = left eye; R/E = right eye; B/E = both eyes; RP = Retinitis pigmentosa; OCT = Optical Coherence Tomography

^a^Individual’s ID as per Fig. 1

### DNA extraction, whole-exome sequencing

Genomic DNA from six members (two affected and four unaffected) of the family, all clinically tested, was extracted [9]. Whole exome sequencing was performed on a 1.0 µg DNA sample of the proband (IV: 3) using Ion Target Seq™ Exome Kit (Applied Biosystems, Thermo Fisher Scientific, Foster City, Carlsbad, California) and Ion Proton™ System (Applied Biosystems, Thermo Fisher Scientific) (In house facility at the GNDU, Amritsar, Punjab).

### Bioinformatics analysis

Sequence reads were aligned to the haploid human reference genome (GRCh37/UCSC hg19) using the Burrows-Wheeler transform algorithm v.0.7.12. Bioinformatics analysis of the WES data in the present study was performed with Ion Reporter Software (criteria as mentioned in the previous study by Goyal et al. [10]).

### Variant validation by Sanger sequencing

Exon 6 of *C8orf37* along with exon-intron boundaries was amplified by PCR, using the following primers Fp-5’-ACAATGAGACTCCTAAAAACAAA-3’ and Rp-5’-GGAACTCCATAATCAAACCTCTA-3’ designed using Primer select (a sub-program of the Lasergene package DNASTAR Inc., Madison, WI). Genomic DNA from another affected individual (IV: 4) and four unaffected family members (III: 1, III: 2, IV: 1, and IV: 2) **(**Fig. 1**)** of the family were amplified and these amplified products were purified and sequenced bi-directionally using BigDye Terminator Cycle Sequencing Kit v3.1 following protocols as mentioned elsewhere [11]. On 3500xL Genetic Analyzer (Applied Biosystems, Thermo Fisher Scientific) (In house facility at the GNDU) electrophoresis was performed. SeqA6 software was used to assemble the sequences, and these were analyzed with the help of SEQSCAPE v3.0 software. Further 107 ethnically matched healthy controls were also tested for the observed variant showing co-segregation with the disease in the analyzed family, to exclude its probability as a polymorphism. Different online bioinformatics software i.e., Human Splicing Finder version 2.4 (http://www.umd.be/HSF*)* and VarCards (http://159.226.67.237/sun/varcards/welcome*)* were applied to know the pathogenicity of the observed nonsense mutation in the present study. VarCards analyze the damaging score of the variant based on at least 13 algorithms. A criterion was set to access the pathogenicity of variants observed in the candidate genes in the present study: (a) known association of the relevant gene with inherited retinal dystrophies (IRDs); (b) interaction with known IRD-associated proteins; (c) variants were considered pathogenic when at least three of the *in-silico* prediction algorithms suggested that a variant is deleterious; (d) pathogenicity was considered according to the American College of Medical Genetics (ACMG) standards and guidelines (as mentioned earlier Goyal et al. [10]). For secondary structure analysis of mutant protein, PSIPred v.3.3 (http://bioinf.cs.ucl.ac.uk/psipred/) and RNA fold web server (http://rna.tbi.univie.ac.at/cgi-bin/RNAWebSuite/RNAfold.cgi) were used.

## Results

Two affected members (IV: 3 and IV: 4) of an arRP family experienced night vision loss since the age of 10 years and 11 years, respectively. Their central vision was preserved and there were no signs of scotoma or dyschromatopsia. Dilated fundus examination of the proband (IV: 3) **(**Fig. 1**)** and another affected sibling (IV: 4) showed typical RP symptoms i.e., attenuation of retinal blood vessels (bilateral), pale optic disc and bone spicule pigmentation at the periphery of the fundus and early macular degeneration. The foveal reflex was dull in both the affected individuals. The cup-to-disc ratio in the proband (IV: 3) has been 0.5 and in his affected sibling (IV: 4) it was 0.3. Fundus examination of the unaffected members (III: 1, III: 2, IV: 1 **(**Fig. 1**)**, and IV: 2) indicated no RP changes **(**Table 1**)**. Optical Coherence Tomography (OCT) examination of both the affected members (IV: 3 **[**Fig. 1**]** and IV: 4) showed the absence of a photoreceptor layer with no signs of macular edema. Additionally, thinning of the central macula has been observed in the affected individual (IV: 3). The ellipsoid zone was disrupted starting from the perifoveal area and extending to the peripheral retina. OCT examination of the unaffected sibling (IV:1) **(**Fig. 1**)** indicated normal retinal architecture. Refractive error in the affected individual IV: 3 was − 4.50D (OD) and − 5.50D (OS) suggesting moderate myopia whereas for other affected individual IV: 4, the refractive error was − 10.00D (OD) and − 10.00D (OS) indicating high myopia. Visual acuity loss in both the affected members was 6/60 in both eyes. Affected individuals IV: 3 (left eye) and IV: 4 (right eye) were also having posterior subcapsular cataract in association with RP. No signs of nystagmus and any extraocular manifestations (especially of Bardet-Biedl Syndrome [BBS]) were observed in both patients **(Supplementary Table 1)**. The family history and the presence of consanguinity indicated autosomal recessive inheritance **(**Fig. 1**)**.

### Whole exome sequencing, filtering, and prioritization of variants

Approximately 12.17GB of raw data was generated by base calling and the panel coverage obtained was 99.89%. 5,81,125 variants were observed in the WES data of the affected individual (IV: 3). Data was analyzed using Ion Reporter™ Software (ver. 5.2) to filter out all the candidate gene variants. Filter for all the previously reported retinal dystrophy genes, homozygosity, and variant effect filter was applied to the WES data. On filtering the variants based on zygosity (homozygous/heterozygous), location (ONTARGET), variMAT score (High), inside gene information, and read depth (> 100), only 16 variants (all in homozygous form) in 12 genes were filtered out **(Supplementary Table 2)** including c.555G > A (p.Trp185Ter) in *C8orf37*. However, based on pathogenicity criteria (as described earlier in methods), *C8orf37* flagged out as the most promising candidate gene. Additionally, all the observed variants except c.555G > A (*C8orf37*) were present in the 1000 Genome database with a minor allele frequency ranging from 19 to 100%. Together, these criteria strongly suggest that the *C8orf37* variant is the one linked with the disease in the present family.

### Variant verification

**Fig. 2:**
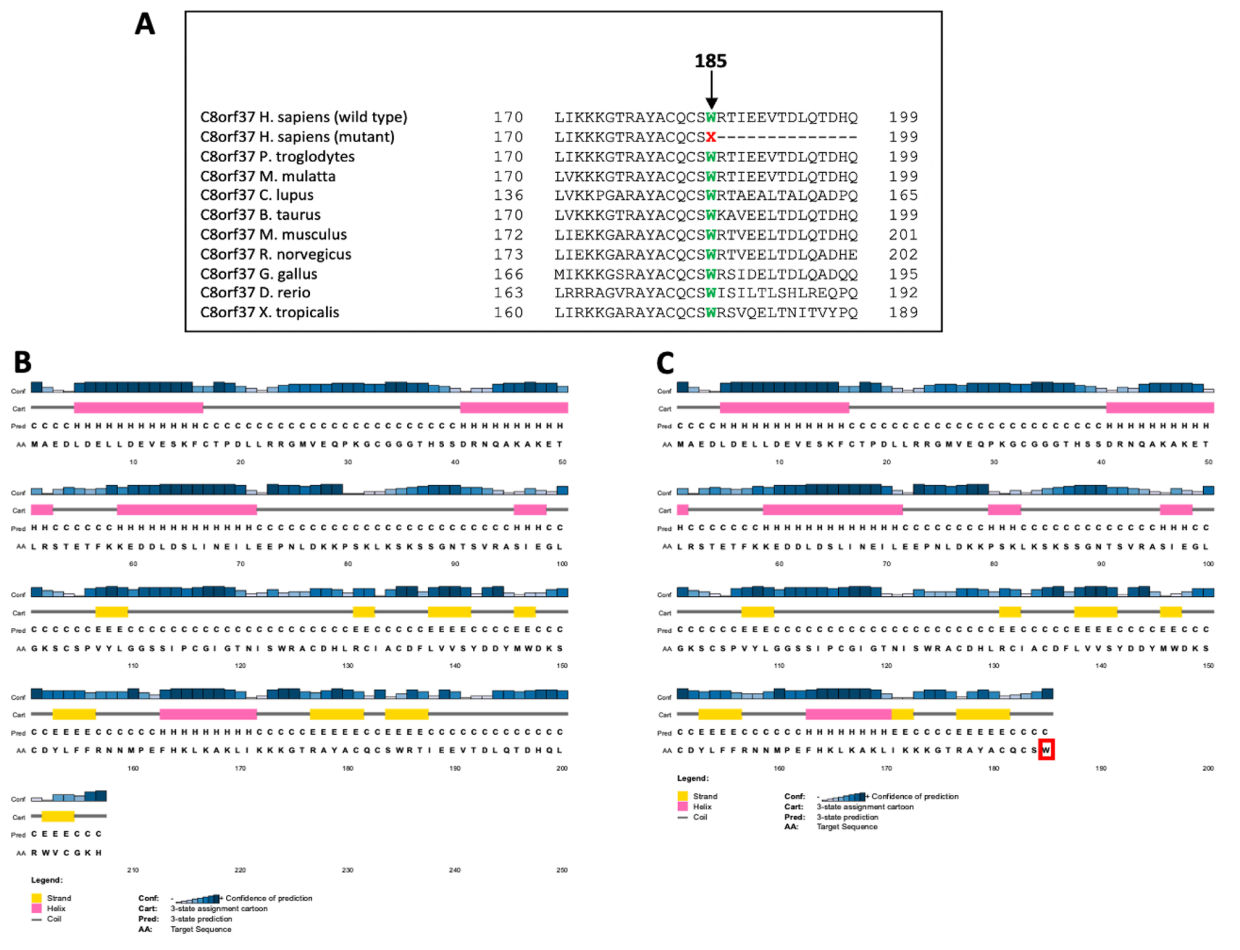
**(A)** Multiple amino acid sequence alignment (NCBI Homologene) of a portion of C8orf37 showing conservation of tryptophan (shown in green) at position 185 as indicated by an arrow in different species. Mutant sequence [*Homo sapiens* (mutant)] in the affected members of the present analyzed arRP-1175 family i.e., replacement of tryptophan by termination codon at position 185 is indicated in red. **(B & C)** Secondary structure prediction of C8orf37 protein sequence by PSIpred in proband IV: 3 with c.555G > A (p.Trp185Ter) mutation. **Section B** represents wild type 207 amino acid protein. **Section C** represents no further formation of protein due to nonsense mutation at 185 amino acid which is highlighted in a red box. H: helix; E: strand; C: coil

Variant c.555G > A (p.Trp185Ter) (rs748014296, https://www.ncbi.nlm.nih.gov/snp/rs748014296) in the *C8orf37* has been previously reported to be associated with arRP in a Pakistani family [12] and RP with early macular degeneration in an arRP family from China [13]. Thus, we proceeded to screen the nucleotide substitution, i.e., c.555G > A (p.Trp185Ter) in the affected sibling of the proband and all the four unaffected members of the family by Sanger sequencing to check its co-segregation with the disease. c.555G > A (p.Trp185Ter) substitution co-segregated with the phenotype in both the affected individuals (IV: 3 and IV: 4) in homozygous form (AA; Fig. 1). Unaffected members of the family (III: 1, III: 2; IV: 1; IV: 2) were heterozygous (GA; Fig. 1) for the observed substitution. Ethnically matched 107 controls, had wild-type sequence in homozygous form (GG). Multiple amino acid sequence alignment (NCBI Homologene) of a portion of C8orf37 indicated tryptophan at position 185 to be highly conserved **(**Fig. 2A**)**. In our results, eight out of 13 functional prediction programs in VarCards showed that c.555G > C (p.Trp185Ter) in *C8orf37* to be deleterious/disease-causing/damaging **(Supplementary Table 3)**, and four predicted the change to be evolutionarily conserved. Human Splicing Finder ver.3.1. (http://www.umd.be/HSF/) predictions indicated that the observed substitution i.e., p.Trp185Ter has probably no impact on splicing.

The protein structure of the *C8orf37* is still not identified and the prediction of the secondary structure of the C8orf37 protein sequence done using PSIpred software (http://bioinf.cs.ucl.ac.uk/psipred/), predicted the loss of functional protein due to truncating mutation at amino acid 185 **(**Fig. 2B and 2C). Moreover, the folding free energy change ∆∆G (which is a difference between ∆G [mutant] and ∆G [wild-type protein]) was − 0.30 kcal/mol, indicating a decrease in the stability of the mutant protein.

## Discussion

In the present study, we investigated a North Indian consanguineous family with two sibs affected with RP in association with early onset maculopathy, posterior subcapsular cataract, and myopia. Both the patients showed c.555G > A nucleotide change in homozygous form. Four unaffected family members were heterozygous for the wild-type allele, and c.555G > A nucleotide substitution was also not observed in 107 ethnically matched controls, nor in the 1000 Genome database (http://www.1000genomes.org/), hence suggestive of excluding it as a polymorphism. The Trp185 residue of C8orf37 is evolutionary conserved, hence indicating tryptophan to be functionally important and c.555G > A (p.Trp185Ter) substitution may have a detrimental effect(s). *C8orf37* (NM_177965.3, NP_808880) localized at chromosome 8q22.1 spans 23.2 kb of genomic DNA and comprises 6 coding exons that encode a 207 amino acids long polypeptide. *C8orf37* encodes a ubiquitously expressed protein with unknown function (contains no known functional domains or motifs) with a predicted molecular mass of ~ 23 kDa. It is highly expressed in the heart, brain, and retina, and the protein co-localizes with polyglutamylated tubulin, located at the base of the primary cilium in human retinal pigment epithelium cells (hTERT-RPE1) and at the base of connecting cilia in mouse photoreceptors [14] (http://www.ncbi.nlm.nih.gov/gene/157657*).* Authors further documented that two third of C-terminal amino acids in C8orf37 are highly conserved from mammals to unicellular flagellates and oomycetes. Héon et al. [15] documented that knockdown of *c8orf37* in zebrafish, resulted in visual impairment along with BBS-specific phenotypes, reduction in the Kupffer’s vesicle, and melanosome transport delay.

Nonsense-mediated decay (NMD) is a quality-control mechanism that lessens the errors in gene expression by targeting mRNA containing premature termination codons (PTC). NMD is reported to be effective when the premature termination codon in the mRNA transcript occurs more than 50–55 nucleotides upstream with respect to the last exon-exon junction [16, 17]. p.Trp185Ter mutation identified in the present study resides in the last exon of *C8orf37* i.e., exon 6, and only 22 amino acids away from the real termination codon, thus it may result in truncated protein or loss of entire protein, in case somehow NMD occurred. The stability of the proteins is critical for their biological function, activity, and regulation of biomolecules. The folding free energy (∆∆G) is a measure of the thermodynamic stability of the proteins. The folding free energy change (∆∆G) for the present nonsense mutation (p.Trp185Ter) has been observed to be -0.30 kcal/mol, indicating less stability of the mutant protein. The C-terminus of C8orf37 is known as the retinal maintenance protein (RMP) domain (as per the Pfam database) and is highly conserved from lower eukaryotes to humans. This RMP domain harbors different missense mutations previously linked with BBS, RP, and cone-rod dystrophy thus indicating the significance of this domain in retina structure and/or functioning. Hence the truncated protein with a loss of 22 amino acids or loss of the entire protein might lead to retinal degeneration in patients in the present analyzed family. Estrada-Cuzcano et al., [14] also reported a nonsense mutation i.e., c.497T > A (p.Leu166Ter) in *C8orf37* in a family with consanguinity and proband had RP and early macular degeneration. The nucleotide substitution c.497T > A (p.Leu166Ter) was in the last exon, 41 amino acids upstream of the real termination codon, and authors predicted mutation resulting in the truncated protein.

To date, 11 different mutations (six missense/nonsense, four splicing, and a single base pair deletion) (http://www.hgmd.cf.ac.uk*)* in *C8orf37* are identified to be linked with either early-onset RP, cone-rod dystrophy or BBS [18]. The identified mutation c.555G > A has previously been reported in two consanguineous arRP families one each from Pakistan [12] and China [13] with two affected siblings in each family. Notably, in the Chinese family, one affected individual carried c.555G > A change in heterozygous form along with a novel hemizygous *OFD1* mutation i.e., c.358 A > G (p.Thr120Ala) and the phenotype being RP with macular degeneration and without any signs of cataract and myopia. Regarding the Pakistani family, authors have reported the clinical examination of only one affected individual (an affected female with ID 863 from family MA13) at three-time points i.e., at the age of 25, 46, and 64, and till her age of 46, the authors have not mentioned anything about the presence of cataract, and moreover, her visual acuity was 6/36. However, the phenotype in the present analyzed family included typical features of RP in association with macular atrophy, posterior subcapsular cataract, and myopia indicating associated ocular anomalies in the second decade of their life as compared to the Pakistani arRP family with the identical mutation. Additionally, visual acuity (VA) loss in the present analyzed family members was worse than that for patients in the Pakistani family. The North Indian family members had < 6/60 visual acuity by the age of 17 and 20 years i.e., at a much younger age, whereas for the Pakistani family the worse VA (< 6/60) has been reported by the age of 64. This variable expressivity in the affected members in the present analyzed family can be attributed to the extensive phenotypic heterogeneity of monogenic diseases and/or due to the presence of the modifiers. Rather than being by chance occurrence of p.Trp185Ter in three different families and all from Asia (one each from Pakistan, China, and India), the Trp185 codon seems to be a mutation hot spot. Rogozin and Pavlov [19] documented that mutation hotspots may result in substitution with similar or even dissimilar residues. Recurrence is considered an important indication that a mutation might be under selective pressure in protein-coding regions. One of the most reliable indicators of a mutation’s driver status is its recurrence in patients. DNA damage and repair processes, on the other hand, do not influence the genome in the same way, and some mutations are more likely than others.

Earlier studies have reported an association of posterior subcapsular cataract and macular degeneration with RP. To date, genetic factors for cataract in RP patients are not known, however, vitreous changes and modifications of the blood-ocular barrier can be the reason for the development of cataract in RP patients (as reviewed by Sahel et al. [3]). Clarke et al. [20] documented that in macular dystrophies, same genes may be responsible for RP and for RP associated with age-related macular degeneration.

## Conclusions

In summary, in the present study, we have observed a recurrent mutation p.Trp185Ter (c.555G > A) in the *C8orf37* in a North Indian family with RP in association with early macular degeneration, posterior subcapsular cataract, and myopia. The identical mutation has already been reported in a Pakistani and a Chinese family with RP and macular degeneration only, indicating phenotypic variability in the present study. The identified mutation might be a common one in Asian arRP cases and can be tested during initial screening in arRP cases belonging to a similar population.

## Electronic supplementary material

Below is the link to the electronic supplementary material.

Supplementary Material 1

Supplementary Material 2

Supplementary Material 3

## Data Availability

The datasets generated and/or analyzed during the current study are available in the BankIt2618771 hg38_knownGene_ENST00000286688.6 OP359018 (https://www.ncbi.nlm.nih.gov/nuccore/OP359018).
