# Supplementary material for: A nonsense mutation in C8orf37 linked with retinitis pigmentosa, early macular degeneration, cataract, and myopia in an arRP family from North India
Source: BMC Ophthalmol. 2023 May 11;23:210. doi: 10.1186/s12886-023-02936-y (PMC10173570; doi:10.1186/s12886-023-02936-y)
Supplement: Supplementary file 1 — Supplementary Material 1 [file 12886_2023_2936_MOESM1_ESM.docx]

**Supplementary Table 1. Phenotypic features of the affected individuals of an arRP (RP-1175) family**

| **Phenotypic features** | **IV: 3** | **IV: 4** |
| --- | --- | --- |
| **RP (age-of-onset)** | Childhood | Childhood |
| **Macular degeneration** | Yes | Yes |
| **Developmental delay** | No | No |
| **Spastic paraplegia** | No | No |
| **Inappropriate social behavior** | No | No |
| **Polydactyly** | No | No |
| **Deafness** | None | None |
| **Renal defects** | None | None |
| **Cognitive impairment** | No | No |
| **Height (cm)*** | 176 | 175 |
| **Weight (kg)*** | 72 | 75 |
| **Body mass index (BMI)** | 23.2  (Normal BMI) | 24.5  (Normal BMI) |
| **Tongue morphology** | Normal | Normal |
| **Dental architecture** | Normal | Normal |

Note: *The weight and height of the affected individuals IV: 3 and IV: 4 were recorded at the age of 20 and 17 years, respectively.
