# Supplementary material for: A nonsense mutation in C8orf37 linked with retinitis pigmentosa, early macular degeneration, cataract, and myopia in an arRP family from North India
Source: BMC Ophthalmol. 2023 May 11;23:210. doi: 10.1186/s12886-023-02936-y (PMC10173570; doi:10.1186/s12886-023-02936-y)
Supplement: Supplementary file 2 — Supplementary Material 2 [file 12886_2023_2936_MOESM2_ESM.docx]

**Supplementary Table 2. Details of variants observed on WES data analysis in the proband (IV: 3) of an arRP family**

| **Sr. No.** | **Chr** | **Gene** | **rs ID** | **cDNA change** | **Protein level change** | **Homozygous/**  **Heterozygous** | **MAF (1000G)** | **Reported phenotype** |
| --- | --- | --- | --- | --- | --- | --- | --- | --- |
| 1. | 7 | *AOAH* | rs59476355 | c.1975dupA | p.Met659AsnfsTer23 | Homozygous | 0.99 | Chronic frontal sinusitis, Acute Ethmoiditis |
| 2. | 7 | *AOAH* | rs57543920 | c.1914dupA | p.Pro639ThrfsTer43 | Homozygous | 0.88 |  |
| 3. | 7 | *ZNF117* | rs1404453 | c.1282C>T | p.Arg428Ter | Homozygous | 0.88 | Ectopic Thymus |
| 4. | 7 | *COL26A1* | rs398095266 | c.1178_1179insG | p.Glu394GlyfsTer47 | Homozygous | 1 | Acute Ethmoiditis, Asthma, Nasal Polyps, Aspirin Intolerance |
| 5. | 7 | *COL26A1* | rs398095266 | c.1184_1185delG | p.Glu396GlyfsTer47 | Homozygous | 1 |  |
| 6. | 7 | *COL26A1* | rs398095266 | c.1184_1185insG | p.Ser397PhefsTer57 | Homozygous | 1 |  |
| **7.** | **8** | ***C8orf37*** | **rs748014296** | **c.555G>A** | **p.Trp185Ter** | **Homozygous** | **NA** | **BBS21, Cone-Rod Dystrophy 16, Autosomal Recessive Retinitis Pigmentosa** |
| 8. | 8 | *STAU2* | rs11419133 | c.351_352insC | p.Ser118LeufsTer35 | Homozygous | 0.99 | NA |
| 9. | 14 | *TRAJ36* | rs869032696 | c.56delC | p.Pro21ProfsTer? | Homozygous | 1 | NA |
| 10. | 19 | *HSH2D* | rs5827321 | c.663delA | p.Lys223SerfsTer131 | Homozygous | 1 | Parietal Foramina |
| 11. | 19 | *HSH2D* | rs5827321 | c.663dupA | p.Ser223AlafsTer6 | Homozygous | 1 |  |
| 12. | 19 | *CARD8* | rs2043211 | c.30T>A | p.Cys10Ter | Homozygous | 0.31 | Inflammatory Bowel Disease 30, Cervical Adenitis |
| 13. | 19 | *SIGLEC12* | rs16982743 | c.85C>T | p.Gln29Ter | Homozygous | 0.19 | Non-Invasive Papillary Urothelial Neoplasm, Bladder Papillary Transitional Cell Neoplasm |
| 14. | 19 | *SBK3* | rs55643341 | c.954dupG | p.Phe319ValfsTer42 | Homozygous | 1 | NA |
| 15. | 20 | *CRNKL1* | rs2273057 | c.98T>A | p.Leu33Ter | Homozygous | 0.52 | Skin Squamous Cell Carcinoma, Skin Melanoma |
| 16. | 22 | *RIBC2* | rs59404993 | c.190_191insC | p.Leu65PhefsTer4 | Homozygous | 0.99 | NA |

ins: insertion; dup: duplication; del: deletion; 1000G: 1000 Genome database; MAF: Minor allele frequency; NA: Not available; BBS: Bardet-Biedl Syndrome
