# Supplementary material for: A nonsense mutation in C8orf37 linked with retinitis pigmentosa, early macular degeneration, cataract, and myopia in an arRP family from North India
Source: BMC Ophthalmol. 2023 May 11;23:210. doi: 10.1186/s12886-023-02936-y (PMC10173570; doi:10.1186/s12886-023-02936-y)
Supplement: Supplementary file 3 — Supplementary Material 3 [file 12886_2023_2936_MOESM3_ESM.docx]

**Supplementary Table 3. Functional predictions for c.555G>A (p.Trp185Ter) substitution in *C8orf37* using different softwares**

| **Algorithm** | **Score** | **Prediction** | **Damaging score criteria** |
| --- | --- | --- | --- |
| LRT | 0.00 | Deleterious | ≤0.001 deleterious; >0.001 tolerable |
| MutationTaster | 1 | Disease_causing | >0.5 disease causing; ≤0.5 tolerable |
| CADD | 44 | Damaging | >20 damaging; ≤20 tolerable |
| DANN | 0.996 | Damaging | ≥0.99 damaging; <0.99 tolerable |
| FATHMM_MKL | 0.977 | Damaging | >0.5 damaging; ≤0.5 tolerable |
| Eigen | 0.826 | Damaging | ≥0 damaging; <0 tolerable |
| GenoCanyon | 1.00 | Damaging | >0.999 damaging; ≤0.999 tolerable |
| fitCons | 0.638 | Tolerable | >0.7 damaging; ≤0.7 tolerable |
| GERP++ | 5.42 | Conserved | ≥2 DNA sequence is conserved |
| phyloP | 7.239 | Conserved | >2 DNA sequence is conserved |
| phastCons | 1.00 | Conserved | >0.999 DNA sequence is conserved |
| SiPhy | 19.219 | Conserved | ≥12 DNA sequence is conserved |
| ReVe | 0.770 | Damaging | ≥0.4 is damaging; <0.4 tolerable |
